# Programmed Death-1(PD-1), a correlate of protection against disease progression in HIV-1 infected long-term non-progressors

**DOI:** 10.1186/1742-4690-9-S2-P254

**Published:** 2012-09-13

**Authors:** J Kyosiimire-Lugemwa

**Affiliations:** 1 MRC/UVRI Uganda Research Unit on AIDS, Kampala, Uganda

## Background

Long-Term Non-Progressors (LTNP) control HIV-1 disease progression but correlates of control are not clear. T-cell activation and expression of Programmed Death-1 (PD-1), a marker of T-cell inhibition/exhaustion, have been suggested as markers of progression to AIDS. We assessed levels of T-cell activation and PD-1 in LTNP and Rapid progressors (RP).

## Methods

We recruited 15 LTNP and 15 RP originally enrolled in the Entebbe Cohort in Uganda. All were ART naïve and 29 were women. HLADR , CD38 and PD-1 levels were assessed in CD4, CD8 and CD45RA T-cells by flow cytometry. HIV-1 disease progression markers: plasma lipopolysaccharide (LPS) levels, HIV-1 RNA viral load (VL), HIV-1 pro-viral DNA load (PVL) and CD4 counts at enrollment were quantified. Comparisons between groups were performed using the Mann-Whitney U test and correlations by Spearman’s linear correlation coefficients.

## Results

Activated (HLADR+CD38+) CD4+CD45RA+ were higher in the LTNP (median 0.64% for LTNP and 0.18% for RP, p=0.03). PD-1 expression in the CD4 and CD8 T-cell subsets was higher in the LTNP (CD4+PD-1+ median 39.6% for LTNP and 1.0% for RP, p=0.001; CD8+PD-1+ median 60.8% for LTNP and 13.5% for RP, p=0.003). VL (p=0.05), PVL (p=0.03), LPS (p=0.005) were higher in the RP and enrollment CD4 count (p=0.0002) was higher in the LTNP. VL, PVL and LPS positively associated with each other and all negatively associated with enrollment CD4 count. CD4+PD-1+ correlated negatively with VL (rs=-0.40, p=0.03), LPS (rs=-0.38, p=0.04) and positively with enrollment CD4 count (rs=0.36, p=0.05). CD4+CD45RA+HLADR+CD38+ correlated positively with enrollment CD4 counts (rs=0.38, p=0.04). Positive correlations were observed between CD4+CD45RA+/-HLADR+CD38+, CD8+CD45RA+HLADR+CD38+ and CD4+PD-1+ and CD8+PD-1+ T-cells.

## Conclusion

Co-expression of PD-1 and activation markers was higher in the LTNP compared to the RP, contrary to other studies. PD-1 correlated with markers of protection against HIV-1 disease progression, suggesting a beneficial role for PD-1.

